# Influence of Oxygen Tension on Dopaminergic Differentiation of Human Fetal Stem Cells of Midbrain and Forebrain Origin

**DOI:** 10.1371/journal.pone.0096465

**Published:** 2014-05-02

**Authors:** Christina Krabbe, Sara Thornby Bak, Pia Jensen, Christian von Linstow, Alberto Martínez Serrano, Claus Hansen, Morten Meyer

**Affiliations:** 1 Department of Neurobiology Research, Institute of Molecular Medicine, University of Southern Denmark, Odense C, Denmark; 2 Department of Molecular Biology and Center of Molecular Biology Severo Ochoa, University Autonoma Madrid.-C.S.I.C. Campus Cantoblanco, Madrid, Spain; 3 The Wilhelm Johannsen Centre for Functional Genome Research, Department of Medical Genetics, IMBG, Panum Institute, University of Copenhagen, Copenhagen, Denmark; French Blood Institute, France

## Abstract

Neural stem cells (NSCs) constitute a promising source of cells for transplantation in Parkinson's disease (PD), but protocols for controlled dopaminergic differentiation are not yet available. Here we investigated the influence of oxygen on dopaminergic differentiation of human fetal NSCs derived from the midbrain and forebrain. Cells were differentiated for 10 days *in vitro* at low, physiological (3%) versus high, atmospheric (20%) oxygen tension. Low oxygen resulted in upregulation of vascular endothelial growth factor and increased the proportion of tyrosine hydroxylase-immunoreactive (TH-ir) cells in both types of cultures (midbrain: 9.1±0.5 and 17.1±0.4 (P<0.001); forebrain: 1.9±0.4 and 3.9±0.6 (P<0.01) percent of total cells). Regardless of oxygen levels, the content of TH-ir cells with mature neuronal morphologies was higher for midbrain as compared to forebrain cultures. Proliferative Ki67-ir cells were found in both types of cultures, but the relative proportion of these cells was significantly higher for forebrain NSCs cultured at low, as compared to high, oxygen tension. No such difference was detected for midbrain-derived cells. Western blot analysis revealed that low oxygen enhanced β-tubulin III and GFAP expression in both cultures. Up-regulation of β-tubulin III was most pronounced for midbrain cells, whereas GFAP expression was higher in forebrain as compared to midbrain cells. NSCs from both brain regions displayed less cell death when cultured at low oxygen tension. Following mictrotransplantation into mouse striatal slice cultures predifferentiated midbrain NSCs were found to proliferate and differentiate into substantial numbers of TH-ir neurons with mature neuronal morphologies, particularly at low oxygen. In contrast, predifferentiated forebrain NSCs microtransplanted using identical conditions displayed little proliferation and contained few TH-ir cells, all of which had an immature appearance. Our data may reflect differences in dopaminergic differentiation capacity and region-specific requirements of NSCs, with the dopamine-depleted striatum cultured at low oxygen offering an attractive micro-environment for midbrain NSCs.

## Introduction

Parkinson's disease (PD) is an incurable neurodegenerative disorder affecting approximately 1% of the population over 60 years of age. The disease is associated with a progressive loss of midbrain dopaminergic neurons in *substantia nigra pars compacta* followed by a coherent depletion of striatal dopamine (DA). Cardinal symptoms include rigidity, tremor, bradykinesia and postural instability, but non-motor symptoms also occur [1].

A number of explorative studies using human fetal, ventral mesencephalic (VM) dopaminergic neurons have shown that intrastriatal transplantation may become an effective future treatment for patients with PD [2]–[5]. However, the use of human fetal tissue is compromised by ethical concerns, suboptimal survival and integration of grafted DA neurons, development of graft-induced dyskinesias in some patients as well as practical problems and logistics related to the procurement and storage of human donor tissue [6]–[10].

Pre-differentiated human embryonic or somatic stem cells represent a potential alternative source of cells for cell replacement therapy in PD [11]. Neural stem cells (NSCs) are proliferative, multipotent cells that can be isolated from specific regions of the developing and mature central nervous system (CNS). Such cells may have significant advantages compared to human fetal VM tissue as they can be propagated to almost unlimited numbers of relatively homogenous cells *in vitro* and frozen without significant loss of cell viability. Nevertheless, an efficient protocol for controlled generation of transplantable and functional dopaminergic neurons is still not available.

Oxygen levels have important effects on cell proliferation, differentiation and survival. Almost all cells, including those of the CNS can sense and respond to changes in oxygen tension. Fine-tuning of oxygenation is of particular interest for cell viability and function as both hyperoxia [12] and hypoxia [13] increase the generation of reactive oxygen species ROS by mitochondria and other cellular oxidant-generation systems potentially leading to activation of cell death programs.

In the normoxic brain, oxygen levels vary from 0.5% in the midbrain to about 8% at *pia mater* [14], [15], but in the stem cell niche of the developing embryo, the oxygen level may be considerably lower [16]. Despite this, most CNS precursor cells are cultured at the non-physiologically high, atmospheric oxygen tension of approximately 21%. Interestingly, a growing body of evidence has revealed that culturing embryonic and NSCs at low oxygen tension (3%) not only increases cell proliferation and survival but also stimulates the dopaminergic differentiation capacity of the cells [17]–[25].

The transcription factor Hypoxia Inducible Factor-1 (HIF-1) is involved in the cellular response to low oxygen, and it acts as an important regulator of glycolysis, mitochondrial oxygen consumption, erythropoiesis, angiogenesis, and cell survival [26]. HIF-1, is a heterodimer consisting of an oxygen regulated alpha-subunit and an oxygen independent beta-subunit [27]. During hypoxic conditions HIF-1alpha stabilizes with HIF-1beta and binds to specific regions in the DNA, triggering the transcription of several downstream targets of HIF-1. These targets include erythropoietin (EPO) and vascular endothelial growth factor (VEGF), which have been found to be involved in neurogenesis both *in vivo* [28], [29] and *in vitro* [29], [30]. VEGF and EPO have furthermore been found to exert neuroprotective effects on midbrain dopaminergic neurons [29], [31]–[33].

In the present study we compared the effect of low versus high oxygen tension on proliferation, survival, and dopaminergic differentiation of two different human NSC lines, one from fetal midbrain and one from fetal forebrain. In addition, we investigated the survival, migration and dopaminergic differentiation of human midbrain and forebrain NSCs after microtransplantation into dopamine-depleted striatal brain slice cultures as a simple *in vitro* model of cell replacement.

## Materials and Methods

### Ethics statement

Human tissues were donated for research after written informed consent of the women seeking abortion. Tissue procurement was performed in accordance with the Declaration of Helsinki and in agreement with the ethical guidelines of the Network of European CNS Transplantation and Restoration (NECTAR). Approval to use these tissues for research was granted by the Lund University Hospital Ethical Committee, and their use was in compliance with Spanish law 35/1988 on Assisted Reproduction. Ethics statements about the human fetal origin of the cells used in the present study can be found in the original reports describing the cell lines [34]–[37].

The animals, housed at Biomedical Laboratory, University of Southern Denmark, were euthanized according Danish and European legislation by authorized staff, in approved facilities (J.nr. 2013-15-2937-00012, Danish Animal Experiments Inspectorate). All relevant procedures were approved by the Animal Research Ethics Committee, Denmark (Dyreforsøgstilsynet; permit No: 2008/561-1523).

### Culturing and passaging of stem cell lines

Cell isolation and immortalization are described elsewhere [34]–[37]. Briefly, human forebrain and ventral mesencephalic (VM) cells were derived from embryos of 10 weeks (Lund University Hospital, Sweden). Immortalization was carried out by infection with a retroviral vector coding for *v-myc* (LTR-vmyc-SV40p-Neo-LTR; replication defective). Derivatives of the resulting cell lines (hNS1 and hVM1 cells) were used for stable retroviral overexpression of Bcl-X_L_ (LTR- Bcl-X_L_-IRES-rhGFP-LTR), essentially as described by [38]. After infection, cells were selected by fluorescence-activated cell sorting (FACS), resulting in the stem cell lines hNSbcl-x_L_ (forebrain) and hVMbcl-x_L_ (midbrain) [34], [39]. Only cells overexpressing Bcl-X_L_ were used since our previous studies have shown that these cells are much more viable and moreover have a higher dopaminergic differentiation capacity as compared to their non-Bcl-X_L_ overexpressing counterparts [18], [34], [39].

Cells were propagated in poly L-lysine (PLL, 10 µg/ml; Sigma) coated culture flasks containing HNSC100 medium (DMEM/F12 w. Glutamax (Gibco), 2% (v/v) 30% glucose (Sigma), 0.5% (v/v) 1 M Hepes (Gibco), 2.5% (v/v) AlbuMAX-I (Gibco), 1% (v/v) N2 supplement (Gibco), 1% (v/v) NEAA (Sigma) and 1% penicillin/streptomycin (Gibco)) supplemented with 20 ng/ml epidermal growth factor (EGF, R&D Systems) and 20 ng/ml basic fibroblast growth factor (bFGF, R&D Systems) at 36°C in controlled atmospheric conditions of 5% CO_2_ and 95% humidified air (20% O_2_, high oxygen group) or 5% CO_2_ and 92% N_2_ (3% O_2_, low oxygen group; monitored by an O_2_-sensitive alarm system; Forma Scientific Inc.). Medium was changed every third day, and cells passaged at 80% confluence.

For cell passaging, medium was removed, adherent cells washed with phosphate buffered saline without calcium and magnesium (D-PBS; pH 7.4; Gibco) and cells detached by trypsin-EDTA (Gibco) for 3-5 min at 36°C. Trypsinized cells were separated using a sterile Pasteur pipette and the resulting cell suspension centrifuged for 5 min at 4°C at 130 × g. The supernatant was removed, and the cells resuspended in new T75 culture flasks before medium change.

### Cell analysis during proliferation

Proliferating forebrain and midbrain NSCs (passage 21–27) were analysed using a handheld automated cell counter (Scepter 2.0; Millipore) and 40 µm sensors (Millipore). After trypzination and resuspension, 100 µl cell suspension from each cell line was measured with the Scepter automated cell counting system. Datasets on size distribution estimated as the number of counts in each dedicated size span were processed with the Scepter software 2.0 (Millipore).

Proliferating forebrain and midbrain NSCs were plated onto PLL coated 24-well or 6-well plates (Nunc) at a density of 10,000 cells/cm^2^ and grown in HNSC100 medium with 20 ng/ml EGF and 20 ng/ml bFGF for 4 days before fixation or analysis using the NucleoCounter NC200 (Chemometec, Allerød, DK). Cells were grown at 36°C at either high (20%) or low (3%) oxygen tension. Cells grown in 24-well plates were fixed and immunostained as described later. The NucleoCounter was used to obtain total cell numbers and cell viability. In brief, medium was removed from 6-well plates, adherent cells washed in D-PBS followed by addition of 500 µl/well Trypsin-EDTA for 3-5 min at 36°C. Detached cells were transferred to individual Eppendorf tubes containing 1 ml HNSC100 medium and immediately analyzed by the NucleoCounter according to the manufacturer's instructions.

### Neuronal differentiation protocols

Human forebrain and midbrain NSCs (passage 21–27) were differentiated for 10 days according to the CK4 protocol in which molecules known to enhance the dopaminergic differentiation are added to the medium in a sequential manner [18]. Briefly, cells were plated onto PLL coated culture trays (Nunc) at a density of either 5,000 or 10,000 cells/cm^2^ and differentiated in HNSC100 medium with 50 ng/ml fibroblast growth factor 8 (FGF8; R&D Systems) for the first three days and then in medium with 25 µM Forskolin (Sigma), 5 ng/ml glial cell line-derived neurotrophic factor (GDNF; Promega) and 25 ng/ml sonic hedgehog (Shh; R&D Systems) for the following 7 days before fixation. Cells were grown at 36°C at either high (20%) or low (3%) oxygen tension, with half of the medium being changed every third day.

### Preparation of striatal slice cultures

Striatal slice cultures were prepared as previously described with a few modifications [40]. In brief, newborn C57BL/6 mice (postnatal day 0–1) were quickly decapitated and the brains isolated under aseptic conditions. After removal of the meninges, brains were placed on their ventral surfaces and cut into 400 µm coronal slices using a McIlwain tissue chopper (Mickle laboratory, Cambridge, UK). The tissue was immediately transferred to Gey's Balanced Salt Solution (Gibco) supplemented with 5% glucose (Merck) for subsequent slice separation and removal of neocortical and subventricular tissue. Striatal slices were placed on semiporous membranes (pore size 0.4 µm, Millipore, Bedford, MA, USA; 4 slices/membrane) and placed as inserts in six-well culture trays with 1 mL serum-containing culture medium per well. The medium was composed of 25% heat-inactivated horse serum (Gibco), 25% Hanks balanced salt solution (HBSS; Gibco), 25 mmol/L -glucose (Merck), 1% penicillin/streptomycin in OPTIMEM (Gibco). The slice cultures were grown at either high (20%) or low (3%) oxygen tension.

### Microtransplantation of pre-differentiated human NSCs into striatal brain slices

Microtransplantation of NSCs was performed using one-day-old striatal slice cultures. Prior to microtransplantation forebrain and midbrain NSCs were pre-differentiated according to the CK4 protocol for 4 days [18]. After trypsination, cells were injected into the central parts of the striatal slice cultures (1000 cells in a 0.1 µl deposit) using a Hamilton microsyringe (Hamilton, Bonaduz, Switzerland) and a micromanipulator (Diesella, Kolding, Denmark). Three days after microtransplantation, the medium was changed to Neurobasal medium (Gibco) added 2% B-27 supplement (Gibco) and 0.5% L-Glutamine (Gibco). Medium was changed twice a week and at day 13 after microtransplantation cultures were fixed and processed for histology.

### Cell fixation and immunochemistry

NSCs and striatal slice cultures were fixed in 4% paraformaldehyde (PFA; Fluka) in 0.15 M phosphate buffer, pH 7.4. For gamma-aminobutyric acid (GABA) staining, cells were fixed in 4% PFA/0.05% glutaraldehyde/0.15 M phosphate buffer.

For immunocytochemistry, cultures were rinsed with 0.05 M Tris-buffered saline (TBS)/0.1% Triton-X-100 (Sigma), then preincubated with TBS/10% donkey or sheep serum (Gibco) according to the host of the secondary antibody, and incubated with primary antibodies diluted in TBS/10% donkey or sheep serum for 24 hrs at 4°C. Antibodies were used at the following concentrations: Beta-tubulin III (β-tub III, mouse anti-; Sigma) 1∶2000; human nuclei (HN; mouse anti-; Chemicon) 1∶500; tyrosine hydroxylase (TH; rabbit anti-; Chemicon) 1∶1200/1∶500; nestin (mouse anti-; BD-Pharmingen) 1∶2000; microtubule-associated protein 2ab (MAP2; mouse anti-; Sigma) 1∶2000; glial fibrillary acidic protein (GFAP; rabbit anti-; DAKO) 1∶5000; active Caspase 3 (rabbit anti-; R&D Systems) 1∶5000; Ki67 (mouse anti-; BD Pharmingen) 1∶500. Control staining was performed by excluding primary antibodies or using rabbit IgG (DAKO) 1∶20,000 and mouse IgG_1_ (DAKO) 1∶200.

Cultures were rinsed in TBS/0.1% Triton-X-100 and incubated for 1 h with biotinylated secondary anti-rabbit or anti-mouse (Amersham) antibodies 1∶200 diluted in TBS/10% donkey or sheep serum. After rinsing in TBS/0.1% Triton X-100 cultures were incubated for 1 h with horse radish peroxidase (HRP) conjugated Streptavidin (DAKO) diluted 1∶200 in TBS/10% donkey or sheep serum. Cultures were then rinsed in TBS before visualization with 3.3′-diaminobenzidine (DAB; Sigma). Cultures were mounted with glass coverslips using Aquatex (VWR) and images were recorded using a Leica DC camera attached to a Zeiss Axiophot microscope.

Striatal slice cultures were immunostained (wholemount) using the procedure described above with few exceptions. Slice cultures were incubated for two days in primary antibodies and two hrs in secondary antibodies and streptavidin. After staining, cultures were transferred to Superfrost Plus glass slides, dehydrated in 99% ethanol, cleared in xylene and mounted with DePeX (VWR).

Double immunofluorescence staining of NSCs and slice cultures was performed essentially as described above either in 24-well plates containing coverslips (NSC cultures) or as wholemount (slice cultures). After preincubation with TBS/5% goat serum, cultures were incubated for 24 hrs (primary cultures) or 48 hrs (slice cultures) with a mixture of two of the following antibodies: HN (mouse anti-; Chemicon) 1∶500; Ki67 (rabbit anti-; Neomarkers) 1∶500; nestin (rabbit anti-; AbD serotec) 1∶1000; GFAP (rabbit anti-; DAKO) 1∶5000; β-tub III (mouse anti; Sigma) 1∶2000; β-tub III (rabbit anti-; Sigma) 1∶2000; MAP2 (mouse anti-; Sigma) 1∶2000; TH (rabbit anti-; Chemicon) 1∶600; TH (mouse anti-; Chemicon) 1∶600; gamma-aminobutyric acid (GABA; rabbit anti-; Chemicon) 1∶500 in TBS/5% goat serum. Subsequently, cultures were incubated with a mixture of Alexa Fluor 555 conjugated anti-mouse IgG and Alexa Fluor 488 conjugated anti-rabbit IgG at 1∶200 for two hrs (primary cultures) or three hrs (slice cultures) at room temperature. Cell nuclei were counterstained with 4′,6-diamidino-2-phenylindole (DAPI; Sigma) at 10 µM in TBS. Cultures were mounted onto glass slides with Prolong Gold mounting medium (Molecular Probes). Images were recorded using a Zeiss Axiophot epifluorescence microscope connected to a Leica DC300 camera or FluoView FV1000MPE - Multiphoton Laser Scanning Microscope (Olympus) and processed using Adobe Photoshop software.

### Western blotting

After trypsination, cell pellets were resuspended in PBS containing Complete Protease Inhibitor Cocktail (Roche Diagnostics) followed by centrifugation at 10,000 rpm for 5 min at 4°C.

Cells were homogenised by tip sonication for 3×10 sec on ice in PBS with Complete Protease Inhibitor Cocktail followed by treatment with PBS/0.1% Triton for 1 h at 4°C followed by centrifugation at 10,000 rpm for 15 min at 4°C. The supernatant was collected and the protein content in all samples was measured according to the Bradford method using the BioRad protein assay (BioRad Laboratories) and bovine serum albumin (Sigma) as the protein standard. A Vmax kinetic microplate reader with SoftMaxPro software was used.

Before electrophoresis, samples were mixed 1∶1 (v/v) with NuPage reducing loading buffer (Invitrogen) and heated to 70°C for 10 min before loading on 4–12% NuPage Bis Tris gels together with SeeBlue Plus prestained standard (Invitrogen). For analysis of TH, β-tub III and GFAP 5 µg, 5 µg and 7.5 µg protein extract was loaded per lane, respectively. Electrophoresis was performed with NuPage MOPS SDS running buffer with 0.25% antioxidant (Invitrogen) for 50 min at 200 V.

Proteins were blotted to PVDF membranes (Immobilon-P; Millipore) for 90 min at 25 V in Transfer buffer (Invitrogen) with 10% Methanol. Membranes were blocked for 1 hr in 5% (w/v) milk powder and 0.05% (v/v) Tween-20 (Merck) in 0.05 M TBS. After repeated washing (0.05 M TBS/0.05% (v/v) Tween-20), the membrane was incubated overnight at 4°C with TH (mouse anti-; Chemicon) 1∶2000; β-tub III (mouse anti-, Sigma) 1∶2000 or GFAP (mouse anti-; Chemicon) 1∶1000 in TBS/Tween-20. Membranes were washed for 4×5 min in TBS/Tween-20 and subsequently incubated with HRP-conjugated rabbit anti-mouse antibody (Dako) 1∶2000 for 1 hr at RT. Membranes were washed before chemoluminiscence development (SuperSignal West Dura Extended substrate; Thermo Scientific) and visualization using a CCD camera (GL2200Pro; Carestream). As loading control all blots were subsequently incubated with alpha-actin antibody (mouse anti-, 1∶8000, Chemicon) over night at 4°C and developed as describes above.

### RNA purification, cDNA synthesis and quantitative real- time PCR

For quantitative real time PCR experiments cultures were grown in triplicates and RNA isolated for each triplicate using RNeasy mini kit (Qiagen). To remove traces of chromosomal DNA, RNeasy on column DNA digestion was performed (Qiagen). RNA quality was assessed by OD260-280 measurements. cDNA was synthesized by use of High Capacity cDNA Reverse Transcription kit (Applied Biosystems) and Q-PCR done as previously described [41]. Primers were designed using Oligo ver. 6 software (Molecular Biology Insights) and ordered from Tag Copenhagen A/S, Denmark. EPO: fwd 5'-AGTGCATGTGGATAAAGCCGTCAGTG-3', rev 5'-CCGGAGAAATTGGAGTAGACTCGAAGA-3'; VEGF: fwd 5'-AAGGAGGAGGGCAGAATCAT-3', rev 5'-ATCTGCATGGTGATGTTGGA-3'; HIF-1alpha: fwd 5'-CCCAGTGAATATTGTTTTTATGTGGATAG-3', rev 5'-GTGGTGGCATTAGCAGTAGGTTCT-3'. The following housekeeping genes were used for normalization: G6PD1A: fwd 5′-CCGGGACAACATCGCCTGCGTTATC-3′,rev 5′-ACGGCTGCAAAAGTGGCGGTGGT-3′; EEF1D1A: fwd 5′-AGAGCCAGAGAGAACATCCAGAAATCCCT-3′; rev 5′-CTCCTCCTCATTGTCACTGCCAAACAG-3′; EIF61A: fwd 5′-AAGTCTTCAGACAGACAGTGGCCGACCAG-3′; rev 5′-ACCACCATCCCAGCAGCAATCACCT-3′. Expression data were calculated as average values from two independent experiments with a maximum number of 12 replicates/group.

### In situ TH enzyme activity

TH enzyme activity was determined for cell cultures by measuring 3,4-dihydroxyphenylalanine (L-dopa) accumulation in the medium after 4 hrs of incubation at 36°C. The analysis was performed using high performance liquid chromatography of (HPLC) with electrochemical detection as described previously [17], [42]. In brief, cell cultures were pre-incubated for 1 h with 400 µl HBSS (Gibco) with 100 µM decarboxylase inhibitor (3-hydroxybenzylhydrazin HCl, Acros Organics) before adding 400 µl fresh HBSS with decarboxylase inhibitor. After 4 hrs, incubation 100 µl samples were collected in vials containing 50 µl acidic mobile phase (see below) and stored until HPLC analysis. The mobile phase consisted of 0.01 M sodium acetate (Merck), 13 mg/l EDTA (Sigma) dissolved in Milli-Q water and adjusted to pH 3.15 with glacial acetic acid and phosphoric acid (Merck). Samples were directly injected into the HPLC system, and the levels of L-dopa determined using an external L-dopa standard (1 pmol/20 µl), essentially as described elsewhere [40], [43].

### Lactate dehydrogenase (LDH) assay

As a measure of cell death, the content of released LDH was determined at days 4, 6 and 10 in 90 µl medium samples. To each sample was added 10 µl of Tris/NaCl buffer (0.813 M Tris, 2.033 M NaCl; pH 7.2) and samples were stored at −20°C until analysis. The rate of conversion of NADH to NAD^+^ as an indicator of LDH activity was measured by a fully automatic spectrophotometer (Cobas Mira, Hoffmann LaRoche). Twenty µl media samples were added 20 µl pyruvate and 240 µl NADH (both Sigma). The absorbance at 340 nm (37°C) was used as an index of NADH concentration. Before each set of measurements, changes in absorbance of LDH standard solutions (Boehringer Mannheim) were measured for calculation of a standard curve [44].

### MTS cell viability assay

Cell viability was determined with an *in vitro* MTS based kit (CellTiter96Aq_ueous_ One solution; Promega). MTS is cleaved by mitochondrial dehydrogenase in viable cells and forms a soluble formazan product, which can be detected by the use of a spectrophotometer. Cells were differentiated as described above at a density of 5,000 cells/mm^2^ (midbrain cells) and 10,000 cells/mm^2^ (forebrain cells) in flat-bottomed 96-well plates. After 10 DIV, CellTiter96A_ueous_ Reagents were added to the wells according to the manufacture's guidelines. After 4 hrs at 36°C, cell viability was determined by measuring the absorbance at 490 nm against control medium in the linear range of the absorption curve using the Vmax kinetic microplate reader and SoftMaxPro software (Molecular Devices).

### Cell counting and statistical analysis

Quantifications of TH-immunoreactive (-ir) cells (n  =  18–20, three independent experiments), Caspase 3-ir cells (n =  8, two independent experiments), Ki67-ir cells (n  =  10, two independent experiments), MAP2-ir cells (n  =  6) and HN-ir cells (n  =  6–9, two-three independent experiments) in differentiated forebrain and midbrain cell cultures were performed using bright field microscopy (Olympus, Ballerup, Denmark). Only cells displaying an extensive immunostaining with a well-preserved cellular morphology were counted. Quantifications of cells were based on counts performed 16 randomly selected areas per well at 200× magnification using an ocular grid (0.5×0.5 mm^2^). Quantification of cells co-expressing TH and β-tub III cells was performed on randomly taken fluorescence pictures at 200× magnification using a Zeiss Axiophot epifluorescence microscope equipped with a Leica DC300 camera. TH-ir and β-tub III-ir cells were counted on single immunofluorescence pictures and subsequently the co-localizing cells were counted on double immunofluorescence pictures overlaid by use of Adobe Photoshop software. All analyzed TH-ir cells were co-expressing β-tub III. For each group >400 cells were investigated (six cultures/group with a total of 24 pictures).

Counts of TH-ir cells (endogenous: n =  17–19; total: n = 13–34, two independent experiments) and HN-ir cells (9–22, two independent experiments) in microtransplanted striatal slice cultures and non-transplanted control slices were performed using the CAST-grid stereological cell counting system (CAST-grid, Olympus, UK). Counting was achieved by using a ×100 oil immersion lens on a Olympus BH-2 microscope connected to a PC. For cultures receiving midbrain-derived cells an area of 10% was included for analysis, whereas an area of 20% was included for analysis of slice cultures receiving forebrain cells and non-transplanted slice cultures. Cell counts were considered as outliers and excluded when differing more than two standard deviations from the mean (all sample groups followed a Gaussian distribution).

Statistical analyses were performed using Instat, GraphPad Software. Comparisons of numbers of TH, Caspase3, Ki67, MAP2, HN-ir cells, TH/β-tub III co-expression, L-dopa accumulation, MTS reduction, qPCR data and LDH release were performed using parametric unpaired t-tests when data followed a Gaussian distribution and revealed equal standard deviations between groups (Bartletts test). Otherwise, a non-parametric Mann-Whitney test was used. Differences were considered statistically significant at P<0.05 marked by ^*^, P<0.01 marked by ^**^ and P<0.001 marked by ^***^. All values in text and graphs are presented as mean ± standard error of mean (SEM).

## Results

### Proliferative capacity of neural stem cells (NSCs) derived from human midbrain versus forebrain – effects of oxygen tension

**Figure 1:**
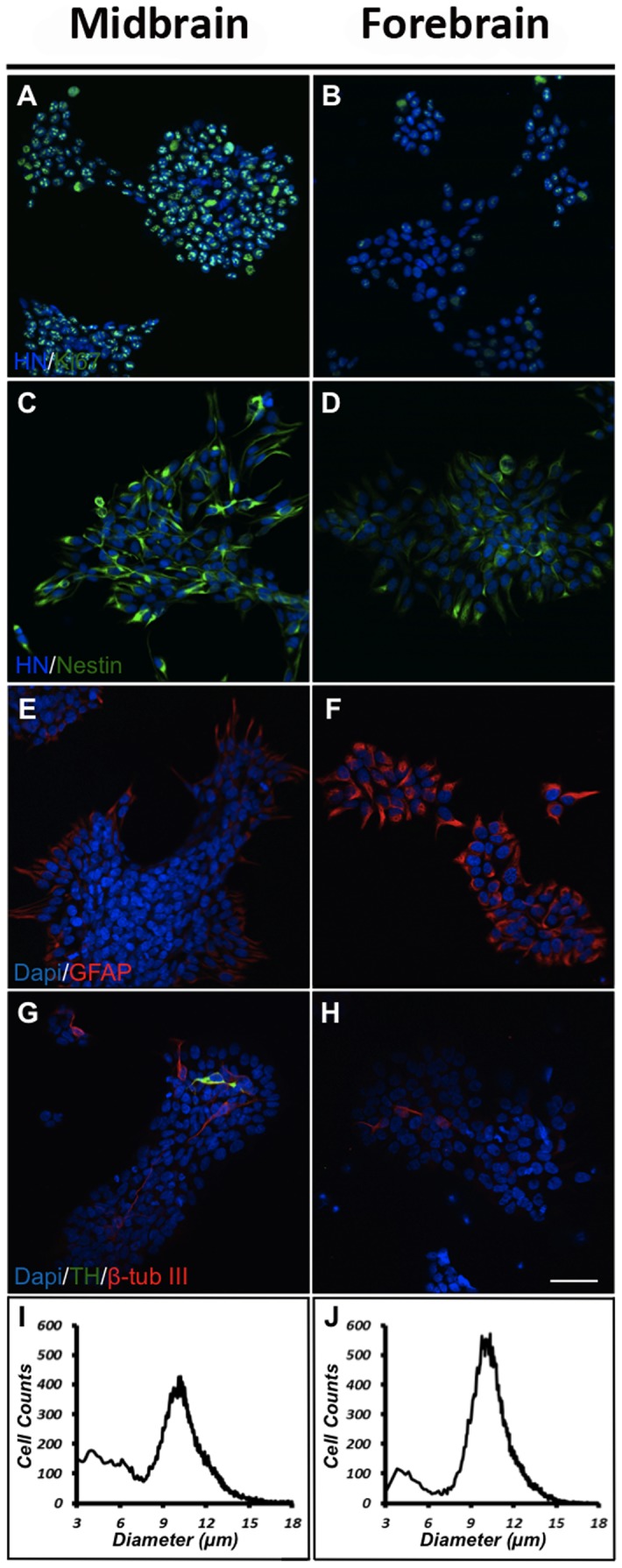
Characterization of proliferating cells. Human midbrain and forebrain neural stem cells (NSCs) were propagated (EGF and bFGF) for 3 days at high oxygen tension (20% O_2_) and immunostained for human nuclei (HN) and the proliferation marker Ki67 (A,B). For both cultures a high density of Ki67-immunoreactive (-ir) cells were seen, although at a higher density in midbrain as compared to forebrain cultures. Most cells in both the midbrain and the forebrain cultures expressed the neural stem cell marker nestin (C,D). Some cells were found to express the astroglial marker glial fibrillary acidic protein (GFAP) in both types of cultures, but clearly at a higher density in forebrain as compared to midbrain cultures (E,F). Few cells in the midbrain cultures were found to express the early neuronal marker β-tubulin III (β-tub III) of which some co-expressed the catecholaminergic marker tyrosine hydroxylase (TH) (G,H). In forebrain cultures only very few of the cells expressed β-tub III, and none of the cells were found to co-express TH (Scale bar  =  50 µm). For cell size estimations midbrain and forebrain cultures were analysed using the Scepter automated counting system. Histograms for both cell populations peaked at approximately 10 µm in diameter (I,J).

To characterize and compare midbrain and forebrain NSCs undergoing expansion, cells were cultured for 4 days *in vitro* (DIV) in medium containing the mitogens bFGF and EGF (high oxygen tension) and immunostained for various markers. The content of dividing Ki67-immunoreactive (-ir) cells was found to be significantly higher for midbrain (87±9% of total cells) compared to forebrain NSCs (52±7% of total cells) (Fig.1 A,B). Cultures were also immunostained for the NSC marker nestin, and as expected most of the cells in both groups were found to express this marker (Fig. 1 C,D). A number of cells in both types of cultures were found to express the astroglial marker glial fibrillay acidic protein (GFAP), although at a higher density in forebrain as compared to midbrain cultures (Fig. 1 E,F). To clarify if any of the cells in the proliferating cultures had spontaneously differentiated into neurons, cells were immunostained for the early pan-neuronal marker β-tubulin III (β-tub III). Whereas some cells in both midbrain and forebrain cultures were found to express GFAP (Fig. 1 E,F), only very few cells stained for β-tub III (Fig. 1 G,H). Cultures were also immunostained for the catecholaminergic marker tyrosine hydroxylase (TH). Very few TH-ir cells were found in midbrain cultures (co-expressing β-tub III), whereas no such cells were seen in cultures derived from forebrain (Fig. 1 G,H). For comparative analysis of cell size, the Scepter automated counting system was used. As can be seen, most NSCs in both types of cultures had a size of approximately 10 µm in diameter (Fig. 1 I,J). The small peak at 3–5 µm reflected noise as similar signals were recorded using cell-free, filtrated D-PBS.

**Figure 2:**
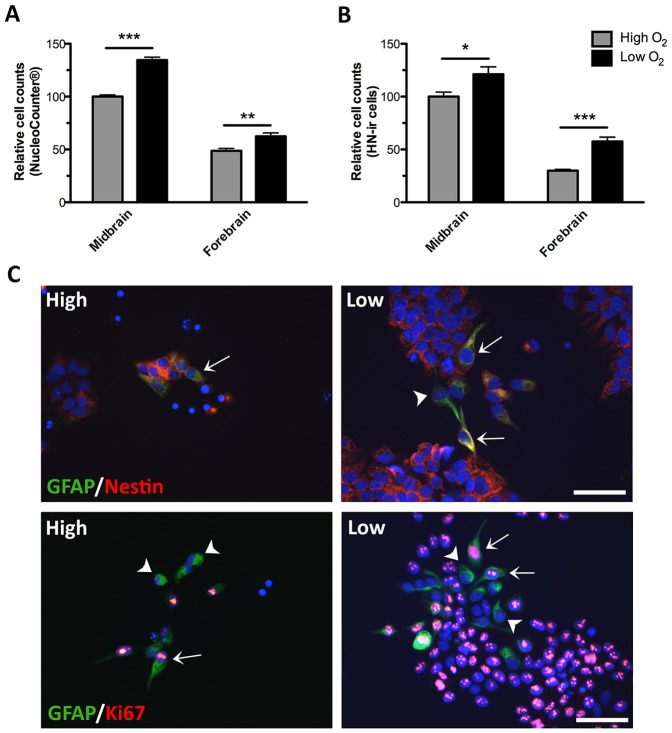
Effects of oxygen on during cell propagation. Human midbrain and forebrain neural stem cells (NSCs) were propagated (EGF and bFGF) at high (20%) or low oxygen tension (3%). At day 4, cells were either trypsinized and the total cell number counted by use of a NucleoCounter (A) or cells were fixed, immunostained for Human Nuclei (HN) and the number of HN-immunoreactive (-ir) cells counted (B). For both midbrain and forebrain NSCs, the total cell numbers were significantly increased when cultures were propagated at low as compared to high oxygen tension. Data are expressed as mean±SEM with midbrain cultures at high oxygen set to 100% (*P<0.05, **P<0.01, ***P<0.001). At day 4, double immunofluorescence staining of forebrain NSCs (high and low oxygen) revealed some (<5%) glial fibrillary acidic protein (GFAP)-ir cells (green) of which a subpopulation co-expressed the neural progenitor cell marker nestin (arrows, upper panel, red) and a subpopulation co-expressed the proliferation marker Ki67 (arrows, lower panel, red). Some GFAP-ir cells did not co-express neither nestin nor Ki67 (arrowheads). Cell nuclei were counterstained with 4′,6-diamidino-2-phenylindole (DAPI) (C). Very few GFAP-ir cells were found in midbrain NSCs cultured at both high and low oxygen tension (not shown). Scale bar  =  50 µm.

To investigate the effect of oxygen on cell proliferation/growth rates, equal numbers of midbrain and forebrain NSCs were propagated (EGF and bFGF) at either high (20%) or low (3%) oxygen tension. At day 4, cells were either dissociated enzymatically (viability 92–96%) and counted by use of a NucleoCounter (Fig. 2A) or cells were immunostained for Human Nuclei (HN) and quantified (Fig. 2B). For both midbrain and forebrain NSCs, the total cell numbers were significantly increased when cultures were propagated at low as compared to high oxygen tension. As it can be seen in Fig. 2AB, cells of midbrain origin in general displayed a higher proliferative activity than cells from the forebrain. This was also confirmed by immunostaining for the proliferation marker Ki67.

As mentioned above, a number of cells in both types of cultures were found to express the astroglial marker GFAP, although at a higher density in forebrain as compared to midbrain cultures (Fig. 1 E,F). Since this protein is also expressed in neural progenitors [45], [46] all the GFAP-ir cells do not necessarily represent astroglial cells but might instead be GFAP expressing NSCs. To investigate this, 4-day old cultures were co-stained for GFAP and nestin, Ki67 or β-tub III by double immunofluorescence staining. Forebrain cultures (Fig. 2C) contained a subpopulation of GFAP-ir cells (green) co-expressing the neural progenitor cell marker nestin (red; upper panel) and some GFAP-ir cells co-expressed the proliferation marker Ki67 (red; lower panel). The relative co-expression of GFAP/nestin and GFAP/Ki67 was similar at high and low oxygen tension. No co-expression of GFAP and β-tub III was observed (not shown).

### Effect of low oxygen tension on dopaminergic differentiation and TH enzyme activity

**Figure 3:**
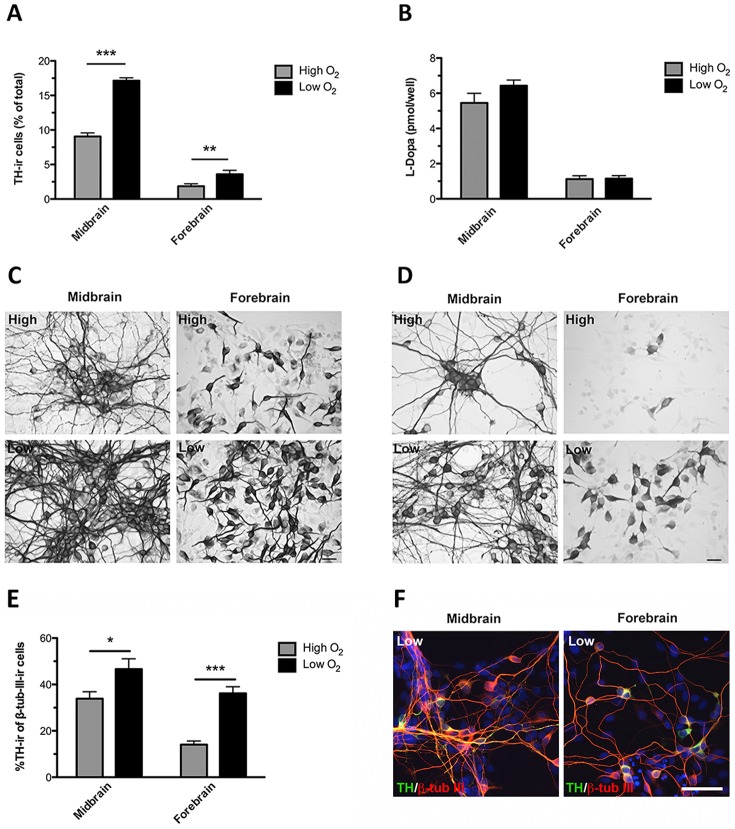
Characterization of tyrosine hydroxylase (TH) and β-tubulin III (β-tub III) expressing cells in cultures differentiated at different oxygen tensions. Cells were differentiated (sequential addition of FGF8, Shh, GDNF and Forskolin) for 10 days at high (20%) or low oxygen tension (3%). The relative contents of TH-immunoreactive (-ir) cells were significantly increased when midbrain and forebrain cultures were grown at low as compared to high oxygen tension (A). Data are expressed as means±SEM (n = 18–20, three independent experiments; ***P<0.001 and **P<0.01). Assessment of TH-activity measured by L-dopa accumulation in the media (B). No significant difference was detected between midbrain or forebrain cultures differentiated at either high or low oxygen tension. The level of accumulated L-dopa in the medium was higher for midbrain cultures as compared to the forebrain cultures, independent of the oxygen tension. Data are expressed as means±SEM (n = 4). Representative microphotographs of cells expressing the early neuronal marker β-tubulin III (β-tub III)-ir in midbrain and forebrain cultures grown at high or low oxygen tension. The density of β-tub III-ir neurons was highest for midbrain cultures, and for both cell types the density of β-tub III-ir neurons was highest at low oxygen (C). Representative microphotographs of TH-ir neurons in midbrain and forebrain cultures grown at high or low oxygen tension (D). Midbrain-derived TH-ir neurons formed small clusters and had distinct cell bodies with long and branching processes, whereas forebrain-derived TH-ir neurons displayed an immature neuronal morphology with relatively short and simple processes. Scale bar  =  20 µm. The relative contents of TH-ir cells of β-tub III-ir neurons were significantly increased when midbrain and forebrain cultures were grown at low compared to high oxygen tension (E). Data are expressed as means±SEM (*P<0.05 and ***P<0.001). Double immunofluorescence staining for TH (green) and β-tub III (red) in midbrain and forebrain cultures grown at low oxygen tension (F). All TH-ir cells were found to co-localize with β-tub III in midbrain and forebrain cultures grown at both high (not shown) and low oxygen tension. Scale bar  =  50 µm.

To investigate the effect of low oxygen tension on dopaminergic differentiation, midbrain and forebrain NSCs were differentiated for 10 DIV according to the CK4 protocol at either high (20%) or low (3%) oxygen tension. Quantification of the relative contents of TH-ir neurons in the cultures revealed a significantly higher percentage for midbrain-derived cells cultured at low oxygen tension as compared to high oxygen tension (P<0.001) (high: 9.1±0.5%; low: 17.1±0.4% TH-ir neurons, mean±SEM, n = 18, three independent experiments) (Fig. 3A). Also the proportion of TH-ir cells in the differentiated forebrain-derived cultures was significantly higher for cultures kept at low as compared to the high oxygen tension (P<0.01) (high: 1.9±0.4%; low: 3.6±0.6% TH-ir neurons, mean±SEM, n = 20, three independent experiments). Furthermore, the yields of TH-ir neurons were higher for midbrain cells than for forebrain cells, regardless of the oxygen tension during their differentiation (Fig. 3A).

TH enzyme activity was determined *in situ* by embedding NSCs, differentiated for 10 DIV, in HBSS with a decarboxylase inhibitor and measuring the accumulation of L-dopa in the medium after 4 hrs of incubation. No significant difference in L-dopa was detected for cultures differentiated at high or low oxygen tension, but midbrain-derived cultures produced significantly (P<0.01) more L-dopa than forebrain-derived cultures grown under identical conditions (Midbrain: high = 5.5±0.6, low = 6.4±0.3; Forebrain: high = 1.1±0.2, low = 1.2±0.2 pmol/l L-dopa, mean±SEM, n = 6) (Fig. 3B).

Comparison of differentiated cultures immunostained for the general neuronal marker β-tub III revealed higher numbers for low oxygen cultures as compared to the high oxygen cultures, an effect observed for both midbrain and forebrain-derived cells. Midbrain β-tub III-ir cells displayed a more complex neuronal appearance with long branched processes compared to a more immature neuronal appearance of forebrain β-tub III-ir cells (Fig. 3C). Similarly, midbrain TH-ir neurons exhibited a complex neuronal morphology with long and branched processes (several multipolar, projection neuron-like cells), whereas forebrain TH-ir neurons produced under identical conditions often had relatively short and unbranched processes (several bipolar, interneuron-like cells) (Fig. 3D). Analyses of the relative contents of TH-ir/β-tub III-ir cells in the cultures revealed a significantly higher percentage for midbrain-derived cells cultured at low oxygen tension as compared to high oxygen tension (P<0.05) (high: 33.9±3.1%; low: 46.6±4.6% TH-ir neurons, mean±SEM, n = 6) (Fig. 3E). Also the proportion of TH-ir/β-tub III-ir cells in the differentiated forebrain-derived cultures was significantly higher for cultures kept at low as compared to the high oxygen tension (P<0.001) (high: 14.1±1.5%; low: 36.2±2.9% TH-ir neurons, mean±SEM, n = 6). Furthermore, the ratios TH-ir cells/β-tub III-ir cells were higher for midbrain cells than for forebrain cells, regardless of the oxygen tension during their differentiation (Fig. 3E). Double immunofluorescence staining for TH and β-tub III in midbrain and forebrain cultures revealed that all TH-ir cells co-localized with β-tub III in cultures grown at both high (not shown) and low oxygen tension (Fig. 3F).

### Effect of low oxygen tension on neuronal differentiation

**Figure 4:**
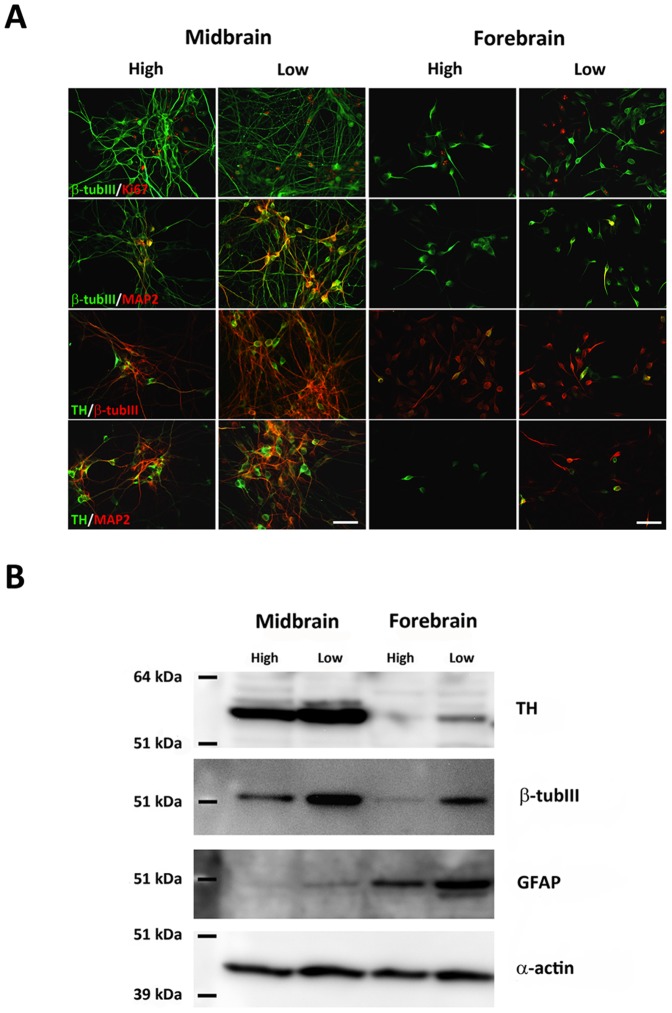
Characterization of differentiated cells. Double immunofluorescence staining was performed for histological characterization of differentiated cultures (A). A small number of cells in both midbrain and forebrain-derived cultures co-expressed the marker of proliferating cells, Ki67, and the early neuronal marker β-tubulin III (β-tub III). The density of β-tub III-ir cells co-expressing the mature neuronal marker microtubule associated protein 2ab (MAP2) was very high for midbrain cultures, particularly when differentiated at low oxygen tension. In forebrain-derived cultures, only few β-tub III-ir/MAP2-ir cells were detected at low oxygen and even fewer at high oxygen. The density of cells co-expressing tyrosine hydroxylase (TH) and β-tub III or MAP2 was highest for midbrain cultures grown at low oxygen, and lowest for forebrain cultures at high oxygen. Scale bar  =  50 µm. Western blotting for TH, β-tub III, and the astroglial marker glial fibrillary acidic protein (GFAP) revealed marked differences depending on both cell origin and oxygen tension (B). The expression of all these markers was higher in cultures differentiated at low oxygen. The level of β-tub III and TH protein was highest for midbrain-derived cells, whereas the highest level of GFAP was detected for forebrain-derived cells.

Double immunofluorescence-stained NSCs differentiated for 10 DIV were used for general characterization of the cellular content in the cultures. Co-expression of Ki67 and β-tub III was seen for a small population of cells in midbrain and forebrain cultures at both oxygen tensions, suggesting that some of the β-tub III-ir cells were still mitotic (Fig. 4A). Many midbrain-derived β-tub III-ir neurons were found to co-express the mature neuronal marker microtubule-associated protein 2ab (MAP2), particularly for cells cultured at low oxygen tension. Quantification of the relative contents of MAP2-ir neurons in the cultures revealed a significantly higher percentage for midbrain-derived cells cultured at low oxygen tension as compared to high oxygen tension (P<0.001) (high: 5.4±0.5%; low: 15.8±1.0% MAP2-ir neurons, mean±SEM, n = 6). Less than 1.0% MAP2-ir neurons were found in forebrain cultures. Some co-expression of β-tub III and MAP2 was seen in forebrain cultures grown at low oxygen tension, whereas only very few β-tub III/MAP2-ir cells were seen at high oxygen tension. Co-expression of TH and β-tub III was found in all groups, although the density of cells co-expressing these to markers was higher in midbrain cultures. To investigate whether the different oxygen levels had affected maturation of the TH-ir cells, cultures were stained for co-expression of TH and MAP2. At both oxygen tensions the density of TH/MAP2-ir cells were much higher in the midbrain as compared to the forebrain cultures. For both cell lines, the density of TH/MAP2-ir neurons increased when cultures were differentiated at low as compared to high oxygen tension, indicating that low oxygen has important effects on the maturation of TH-ir cells. For midbrain-derived cultures, the density of TH/MAP2-ir cells was high at both oxygen levels (high/low), whereas only very few cells co-expressed these two markers in forebrain-derived cultures (Fig. 4A). No TH and GABA co-expression was seen in differentiated midbrain cultures, whereas some differentiated forebrain cells were found to co-express these markers (data not shown).

Both TH, β-tub III and GFAP expression was also investigated by Western blotting using protein derived from differentiated midbrain and forebrain cell cultures. For both cell lines, the relative content of TH was found to increase when cultures were differentiated at low as compared to high oxygen tension. Bands for TH were more notable for midbrain than forebrain cultures and almost absent in the high oxygen treated forebrain cultures correlating well with the results shown in Fig. 3A. The relative contents of β-tub III and GFAP were also increased when cells were cultured at low oxygen as compared to high oxygen tension. However, whereas Western blotting for β-tub III gave rise to the highest signal intensity for midbrain-derived cells, bands of much higher signal intensities were detected for GFAP in forebrain cultures as compared to midbrain cultures (Fig. 4B).

### Effect of low oxygen tension on cell death, cell viability and proliferation

**Figure 5:**
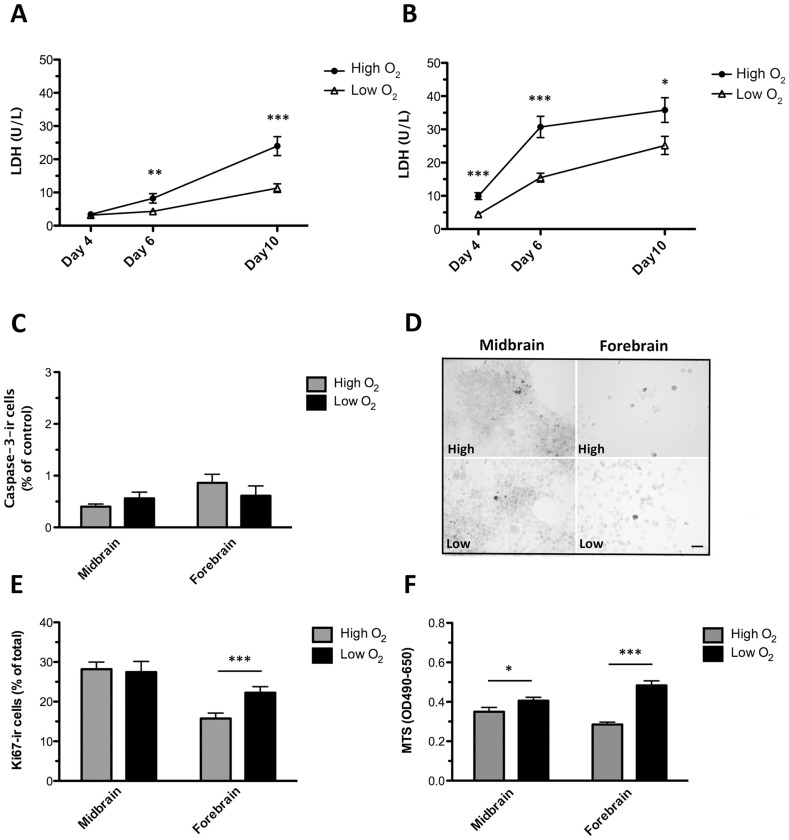
Effects of oxygen on cell death and cell proliferation. Assessment of cell death, cell proliferation and cell viability in midbrain and forebrain NSC cultures differentiated (sequential addition of FGF8, Shh, GDNF, and Forskolin) for 10 days at high (20% O_2_) or low oxygen tension (3% O_2_). Level of lactate dehydrogenase (LDH) in conditioned culture medium from differentiating midbrain (A) and forebrain (B) cells. For both cell types, significantly lower levels of LDH were detected at low oxygen tension. Data are expressed as means±SEM (n = 14–22, three independent experiments; ***P<0.001, **P<0.01, *P<0.05). Quantification of the relative content of active Caspase3 (Casp3)-immunoreactive (-ir) cells in midbrain and forebrain-derived cultures showed no significant differences between oxygen groups (C). Data are expressed as mean±SEM. Representative photomicrographs of active Caspase3-ir cells (D). Densities of Caspase3-ir cells were small for all cultures, and no differences were found between groups. Scale bar  =  50 µm. Quantification of dividing Ki67-ir cells revealed no significant difference for midbrain cells cultured at high and low oxygen, whereas a significantly higher proportion of Ki67-ir cells was found for forebrain cells cultured at low as compared to high oxygen (E). Analysis of MTS reduction showed higher cell viability in midbrain and forebrain cultures differentiated at low as compared to high oxygen tension. Data are expressed as means±SEM (n =  8–10, two independent experiments; ***P<0.001, **P<0.01, *P<0.05).

To address whether the oxygen level affected cell death during differentiation, cultures were differentiated for 10 DIV and the medium collected at days 4, 6 and 10 for analysis of lactate dehydrogenese (LDH). After 4 days, no significant difference in LDH was seen for midbrain cultures, whereas significantly lower LDH levels were detected in medium from forebrain cultures differentiated at low as compared to high oxygen tension (P<0.001). After 6 and 10 days of differentiation, significantly lower levels of LDH were found for both midbrain and forebrain cultures grown at low as compared to high oxygen tension (6 DIV: P<0.01, P<0.001, 10 DIV: P<0.001, P<0.05; midbrain and forebrain, respectively) (Fig. 5A and Fig. 5B, midbrain and forebrain, respectively). Throughout the differentiation, levels of released LDH were higher in conditioned medium from forebrain as compared to midbrain cultures (Midbrain_high_: 4 DIV  =  3.4±0.5, 6 DIV  =  8.2±1.4, 10 DIV  =  24.0±2.8; Midbrain _low_: 4 DIV  =  3.2±0.6, 6 DIV  =  4.3±0.4, 10 DIV  =  11.3±1.3 U/L LDH; Forebrain _high_: 4 DIV  =  9.9±1.0, 6 DIV  =  30.7±3.2, 10 DIV  =  35.8±3.7; Forebrain _low_: 4 DIV  =  4.4±0.8, 6 DIV  =  15.5±1.3, 10 DIV  =  25.2±2.7 U/L LDH; mean±SEM, n  =  14–22, three independent experiments) (Fig. 5A,B).

To further assess cell death, cultures were immunostained for the apoptotic cell marker active Caspase3. For both cell lines, no significant differences in numbers of Caspase3-ir cells were found between the low and high oxygen group (midbrain: high  =  0.4±0.1%, low  =  0.6±0.1%; forebrain: high  =  0.9±0.2%, low  =  0.6±0.2%, mean±SEM; n = 8, two independent experiments) (Fig. 5C,D).

To address whether changes in oxygen tension had influenced the number of cells persisting in a proliferating phase during differentiation, cultures were immunostained for Ki67. The proportion of proliferating cells in midbrain cultures did not differ between low and high oxygen cultures (high  =  28.2±1.8%, low  =  27.5±2.7% Ki67-ir cells, mean±SEM, n = 10, two independent experiments). For forebrain cultures, differentiation at low oxygen tension resulted in a significantly higher percentage of proliferating cells as compared to differentiation at high oxygen (P<0.01) (high  =  15.8±1.4%, low  =  22.3±1.5% Ki67-ir cells, mean±SEM, n = 10, two independent experiments). Looking across the cell lines, the relative proportion of proliferating Ki67-ir cells was higher for midbrain as compared to forebrain cells (Fig. 5E). Evaluation of cell viability was carried out by measuring MTS reduction. MTS is a tetrazolium compound used in colometric assay, and can under defined conditions, be used to determine the number of viable cells in cultures. MTS measurements revealed a significant difference between the low and the high oxygen midbrain cultures (P<0.05) (high =  0.35±0.02, low  =  0.41±0.02 OD490-650, mean±SEM, n = 8) and between forebrain groups (P<0.001) (high: 0.29±0.01, low: 0.48±0.02 OD490-650, mean±SEM, n = 8) (Fig. 5F). The increased MTS reduction (viable cells) in the low oxygen cultures could be caused by a higher number of proliferating cells or less cell death in these cultures.

### Relative expression of genes involved in the cellular response to low oxygen

**Figure 6:**
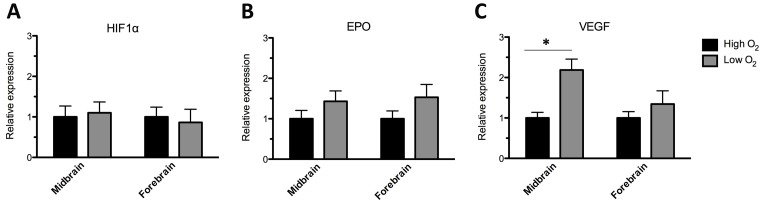
Q-PCR analysis of the expression of hypoxia inducible factor 1alpha (HIF-1α), Erythropoietin (EPO) and vascular endothelial growth factor (VEGF). Q-PCR analysis was performed for midbrain and forebrain cultures differentiated for 10 days *in vitro* (DIV) (sequential addition of FGF8, Shh, GDNF, and Forskolin) at either high (20%) or low (3%) oxygen tension. Analysis of HIF-1alpha mRNA revealed no significant difference between midbrain or forebrain cultures differentiated at high or low oxygen tension (A). Analysis of EPO mRNA revealed a strong tendency for a higher level in midbrain and forebrain cultures differentiated at low as compared to high oxygen (B). A significantly higher level of VEGF mRNA was found in midbrain cultures differentiated at low as compared to high oxygen, whereas no significant difference was found between forbrain cultures grown at low as compared to high oxygen tension however, there was a tendency for higher expression of VEGF in the forebrain cultures differentiated at low oxygen (C). Data are expressed as means±SEM (12 replicates/group, two independent experiments, *P<0.05).

To investigate the transcription of three genes known to be involved in the cellular response to low oxygen, mRNA was purified from midbrain and forebrain cultures differentiated for 10 days. The expression of the genes at high oxygen was set to 1, and the relative expression of the genes at low oxygen was calculated (Fig. 6). The analysis revealed no significant difference in the expression of Hypoxia Inducible Factor-1alpha (HIF-1alpha (Midbrain: high  =  1±0.3, low  =  1.1±0.3; Forebrain: high  =  1±0.2, low  =  0.86±0.3; mean±SEM, 12 replicates/group, two independent experiments) or erythropoietin (EPO) (Midbrain: high  =  1±0.4, low  =  1.4±0.7; Forebrain: high  =  1±0.3, low  =  1.5±0.4; mean±SEM, 12 replicates/group, two independent experiments) between midbrain and forebrain cultures differentiated at high or low oxygen tension, although there was a trend for increased expression of EPO when cells were differentiated at low oxygen. Analysis of vascular endothelial growth factor (VEGF) revealed significantly higher expression for low oxygen midbrain cultures as compared to the high oxygen midbrain cultures, whereas no such difference was found for forebrain cultures (high versus low oxygen) although there were a tendency for higher VEGF expression at low oxygen (Midbrain: high  =  1±0.1, low  =  2.2±0.5; Forebrain: high  =  1±0.3, low  =  1.3±0.5; mean±SEM, 12 replicates/group, two independent experiments).

### Microtransplantation of midbrain and forebrain NSC-derived neurons into striatal slice cultures

**Figure 7:**
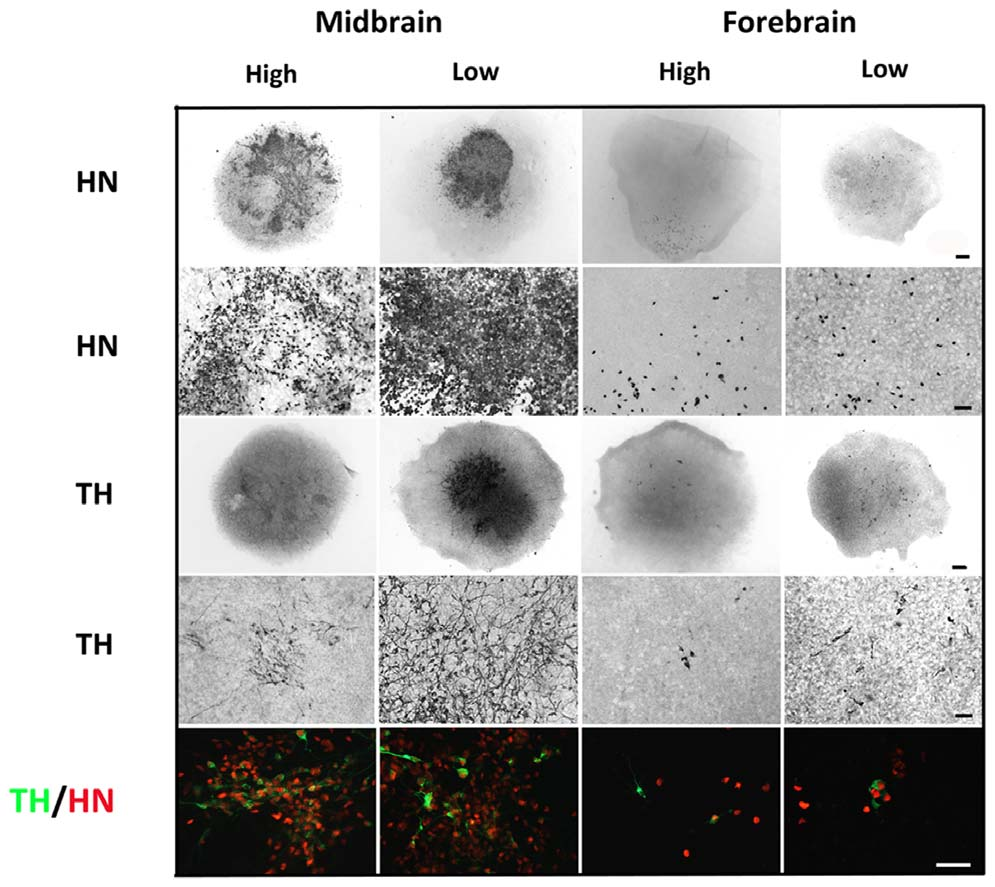
Microtransplantation of human midbrain- and forebrain-derived stem cells into striatal slice cultures. Midbrain and forebrain neural stem cells (NSCs) were pre-differentiated for 4 days *in vitro* (DIV)(sequential addition of FGF8, Shh, GDNF, and Forskolin) and microtransplanted into one-day old mouse striatal slice cultures. Microtransplanted cultures were grown for 13 days at high (20%) or low oxygen tension (3%). Representative photomicrographs of human nuclei (HN)-immunoreactive (-ir) cells. In slice cultures microtransplanted with midbrain-derived NSCs large transplants were seen, whereas slices receiving forebrain-derived NSCs contained HN-ir at a more sparse density. Numerous tyrosine hydroxylase (TH)-ir cells were found in cultures transplantated with midbrain-derived cells, particularly when grown at low oxygen tension, whereas the density of TH-ir cells was relatively low in all grafts of forebrain-derived cells. Midbrain TH-ir cells, especially those cultured at low oxygen, displayed a very mature neuronal morphology with very long and branching processes. In contrast, exogenous forebrain TH-ir cells appeared very immature with very short and simple processes. Double immunofluorescence labeling for TH and HN revealed almost 100% co-expression of these markers in all cultures. Scale bars  = 250 µm (HN and TH above), Scale bars  =  25 µm (HN, TH below and TH/HN).

To investigate whether the cells were transplantable and whether low oxygen tension also affected differentiation of midbrain and forebrain neuronal precursors after micro-transplantation into striatal slice cultures (simplified model of the dopamine-depleted striatum in Parkinson's disease), cells were pre-differentiated for 4 DIV according to the CK4-protocol (sequential addition of FGF8, Shh, GDNF and Forskolin) and seeded onto one-day old mouse striatal slice cultures (1000 cell/culture) and grown at either high or low oxygen tension for 13 days (Fig. 7).

For identification of human midbrain and forebrain cells in the mouse striatal brain slices, cultures were immunostained for human nuclei (HN). For midbrain-derived cells, large grafts were seen, all surrounded by a halo of HN-ir cells that had migrated to the peripheral aspects of the slice cultures.

Quantification of HN-ir cells revealed high numbers of human midbrain cells at both high and low oxygen tension, whereas significantly fewer human forebrain cells were detected at both oxygen tensions (P<0.001). Moreover, the numbers of midbrain and forebrain HN-ir cells were significantly higher at low as compared to high oxygen tension (P<0.001) (Midbrain: high  =  5650±814, low  =  7250±582; Forebrain: high  =  663±138, low  =  1329±206HN-ir cells, mean±SEM, n = 8–22, two independent experiments).

For quantification of human NSC-derived TH-ir neurons in the striatal brain slices, cultures with and without pre-differentiated human NSCs were immunostained for TH. The number of intrinsic (murine) intrastriatal TH-ir cells was then subtracted from the total number of TH-ir cells found in the microtransplanted cultures.

Whereas the density of TH-ir cells in cultures receiving midbrain cells was high, particularly for the low oxygen group, and displayed very long and branching processes, the density of TH-ir cells in slices receiving forebrain cells were low and the TH-ir cells displayed immature neuronal morphology with short and simple processes. TH-ir cells were found throughout striatal brain slices grafted with midbrain-derived cells, while forebrain-derived cells were more restricted to the site of cell placement. To verify that the TH-ir cells were of human origin, cultures were double immunofluorescence stained for TH and HN, and as it can be seen in Fig. 7 almost all TH-ir cells co-expressed HN.

**Figure 8:**
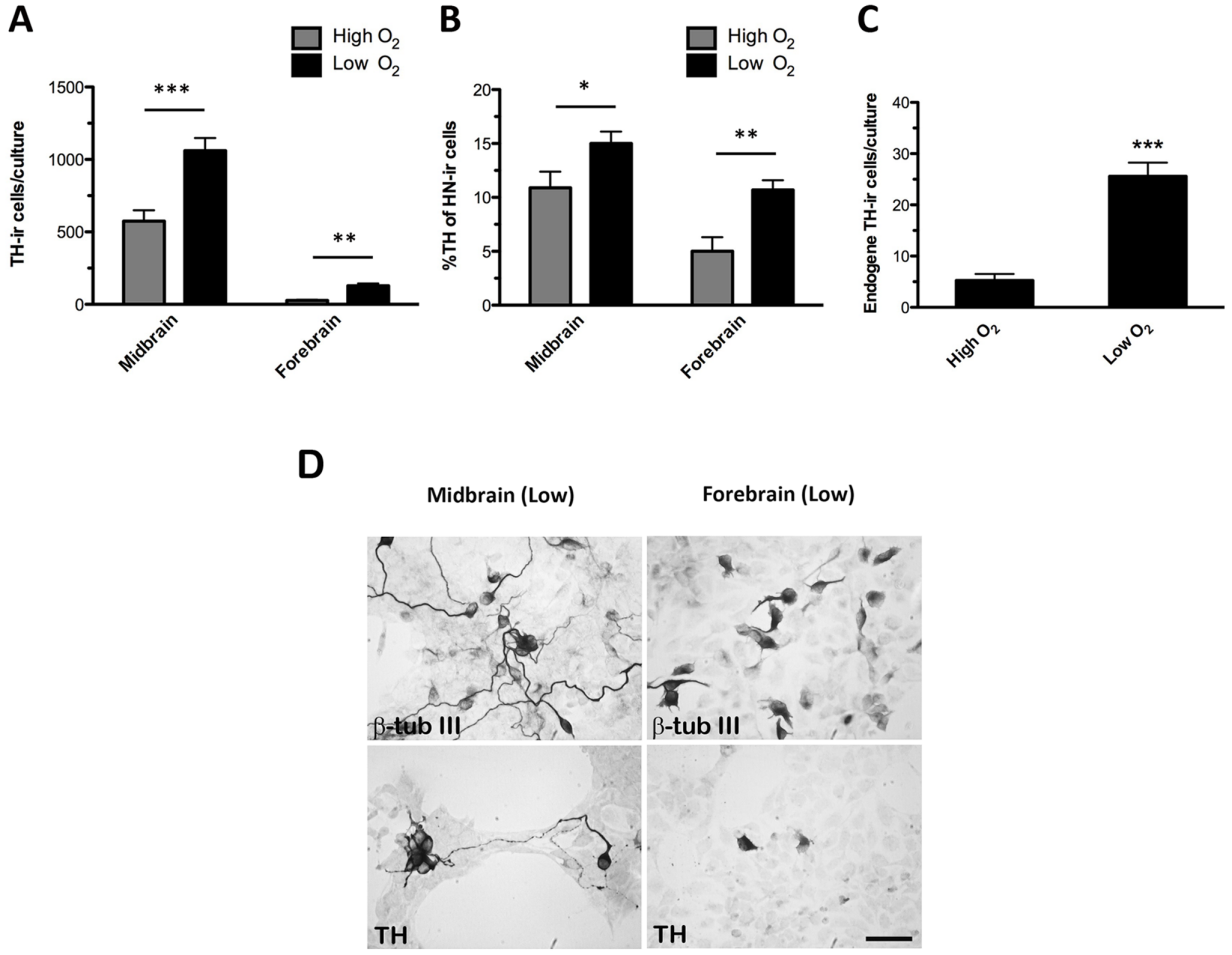
Quantification of exogenous and endogenous tyrosine hydroxylase (TH)-immunoreative (-ir) cells in striatal slice cultures. Pre-differentiated (sequential addition of FGF8, Shh, GDNF, Forskolin) midbrain and forebrain-derived neural stem cells (NSCs) were microtransplanted into mouse striatal slice cultures (1000 cells/slice) and grown for 13 days *in vitro* (DIV) at high (20% O_2_) or low (3% O_2_) oxygen tension. Quantification of the number of TH-ir cells revealed a significantly higher number of TH-ir cells in midbrain or forebrain cultures grown at low as compared to high oxygen tension (A). For both cell lines, the relative proportion of TH-ir cells was significantly higher for cultures grown at low as compared to high oxygen tension (B). The number of endogenous TH-ir cells in non-grafted slice cultures grown for 14 DIV was significantly higher when exposed to low as compared to high oxygen tension (C). Data are expressed as means±SEM (n = 13–34 (TH), two independent experiments; n = 17–19 (endogenous TH), two independent experiments; ***P<0.001,**P<0.01,*P<0.05). Representative microphotographs of midbrain and forebrain adherent cultures pre-differentiated for 4 DIV (D). For both the midbrain and the forebrain cultures, numerous cells expressing the early neuronal marker β-tubulin III (β-tub III) were seen. Few TH-ir cells were found in all cultures, but whereas most midbrain TH-ir cells had started to develop processes, most forebrain TH-ir cells still appeared very immature. Scale bar  =  50 µm.

For the striatal slices microtransplanted with pre-differentiated midbrain NSCs, the total number of human TH-ir cells was found to be significantly higher for low oxygen cultures as compared to the high oxygen cultures (P<0.001) (high  =  575±74, low  =  1061±87 TH-ir cells, mean±SEM, n =  18-31 two independent experiments). Also the number of forebrain-derived TH-ir cells was found to be significantly higher at low as compared to high oxygen (P<0.01) (high  =  26.7±5.7, low  =  128.5±16.3 TH-ir cells, mean±SEM, n =  13–34, two independent experiments). However, the number of TH-ir cells was higher for slices receiving midbrain cells as compared to forebrain cells, independent of the oxygen tension applied (Fig 8A).

For both cell types, the relative contents of TH-ir cells (percent of total cells) were calculated. It revealed that culturing at low oxygen tension resulted in a significantly higher proportion of TH-ir cells (midbrain: P<0.05; forebrain: P<0.01) (midbrain: high  =  10.9±1.5%, low  =  15±1.1%; forebrain: high  =  5±1.3%, low  =  10.7±0.9% TH-ir cells, mean±SEM, n = 13–34) (Fig. 8B). Interestingly, the number of endogenous TH-ir cells was increased by 5 fold (P<0.001) when the striatal brain slices were grown at low as compared to high oxygen tension (high  =  5.3±1.2, low  =  25.6±2.7, n = 17–19, two independent experiments) (Fig. 8C).

For characterization of the pre-differentiated adherent cell cultures, midbrain and forebrain cultures were immunostained for β-tub III and TH. Numerous β-tub III-ir cells were found in both midbrain and forebrain cultures. In the midbrain cultures most β-tub III-ir cells had long processes. None of the forebrain-derived β-tub III-ir cells were found to exhibit a complex neuronal morphology, indicating that these cells were still in an immature state. Also TH-expressing cells were observed in all pre-differentiated cultures, although at a higher number for midbrain compared to forebrain cultures.

Most of the TH-ir cells in the midbrain cultures displayed an immature neuronal morphology, while only a few cells displayed long processes. In forebrain cultures TH-immunostaining was detected in cell bodies, and none of the cells displayed distinct neuronal morphologies (Fig. 8D).

## Discussion

Cell replacement therapy using stem cells holds considerable promise for the future treatment of Parkinson's disease (PD), but a protocol for controlled dopaminergic differentiation is still not available. Here we modeled and compared the influence of oxygen on proliferation, dopaminergic differentiation and survival using human fetal NSCs of midbrain and forebrain origin.

### Characterization of stem cell lines

For characterization of EGF and bFGF treated proliferating stem cells, cultures were immunostained for nestin. Almost all cells expressed this general marker of neural precursor cells, which was in accordance with previous studies showing that both the midbrain stem cell line and the forebrain stem cell line express nestin even after long-term *in vitro* propagation [34], [35], [37], [38], [47]. We also found GFAP expression during propagation but mainly in forebrain-derived cultures. Although, this GFAP expression could reflect cells that had spontaneously differentiated into astrocytes, the staining pattern was more like that seen for nestin-ir cells, and hence speculated to represent GFAP expressing NSCs [45], [46]. This hypothesis was partly supported by our double immunofluorescence staining revealing subpopulations of GFAP/nestin and GFAP/Ki67 co-expressing cells.

A large majority of cells in midbrain-derived cultures (87%) expressed the proliferation marker Ki67, whereas fewer forebrain cells (52%) expressed this marker. Total cell counts obtained at day 4 during proliferation also showed that cells of midbrain origin in general displayed a higher proliferative activity than cells from the forebrain. This supports another study in which midbrain NSCs were found to have a higher proliferating rate than forebrain NSC cells, although this difference was only seen when the NSCs were cultured at low oxygen tension [21]. For both midbrain and forebrain NSCs we found a significantly higher proliferation at low compared to high oxygen tension, which is in agreement with other studies on rodent NPCs [17], [22], [25], [48] and human NPCs [21], [23], [49] and human ESCs [20]. Only very few cells in the cultures had spontaneously differentiated into neurons (β-tub III-ir) of which few were found to co-express the catecholaminergic marker TH. Thus, the very low content of differentiated cells in both types of cultures showed that our cell lines were maintained in an undifferentiated state, corresponding well with other studies showing that neural precursors can undergo long-term expansion *in vitro* using EGF and bFGF [21], [36]–[39], [50].

### Low oxygen tension promotes dopaminergic differentiation of midbrain and forebrain stem cells

For historical reasons, NSCs are often cultured at 36°C in a gaseous atmosphere of 5% CO_2_ and 95% air corresponding to an oxygen tension of about 20%. Whereas the temperature and the CO_2_ imitate the body core temperature and the venous CO_2_ concentrations, respectively, the oxygen tension does not reflect the physiological tissue level of oxygen in the developing and adult human brain, which has been reported to range from <1–8%, depending on the brain region in question [14], [15], [51]. Previous studies have shown that changes in oxygen tension towards more physiological levels stimulate dopaminergic differentiation of rat neural progenitor cells [17], [22], human NSCs/NPCs [18], [21], [34] and human embryonic-derived NSCs [20].

Here we investigated and compared the effects of oxygen tension on dopaminergic differentiation of midbrain and forebrain-derived NSCs by differentiating cultures at either high (20%) or low (3%) oxygen tension. To induce dopaminergic differentiation, cells were treated with FGF8, Shh, GDNF and Forskolin in a sequential manner. This protocol has been found very effectively to promote dopaminergic differentiation of the human midbrain-derived NSCs used in the present study [18].

Here we observed a 1.9-fold increase in the yield of TH-ir cells for both stem cell lines when cultured at low as compared to high oxygen tension. The relative proportion of TH-ir cells was much higher for midbrain cultures as compared to the forebrain cultures, which was confirmed by Western blot analysis. In addition, TH enzyme activity, measured by release of L-dopa, was found to be significantly higher for midbrain-derived cultures. Surprisingly, no significant difference was found in L-dopa release between the high and low oxygen groups. One explanation for this could be that the TH enzyme requires oxygen as a co-factor for full activity [52]. This hypothesis is supported by one of our previous studies, in which we found that rat mesencephalic precursor-derived TH-ir cells released more DA when shifted from low to high oxygen for the last two days of differentiation as compared to those cultured at high oxygen throughout the differentiation period. Western blot analysis of the same cultures revealed no difference in TH-band intensities, indicating that TH-enzyme activity and not TH-enzyme expression was affected by the change of oxygen tension [17]. It is assumed that a similar mechanism is responsible for the lack of difference in L-dopa release observed for cultures grown at high and low oxygen in the present study.

The morphology of the TH expressing neurons also differed among the cell lines. Whereas the TH-ir neurons in the midbrain cultures displayed a mature neuronal morphology with a round cell body and very long and branching processes, most of the TH-ir cells in the forebrain cultures appeared relatively immature. These observations were supported by double-immunolabeling for TH and MAP2, a general marker of mature neurons, showing a high degree of co-localization in midbrain cultures, but not in forebrain cultures, although the extent of co-localization was increased in both the midbrain and forebrain cultures grown at low oxygen tension. This implies that after 10 days of differentiation most of the forebrain-derived TH-ir neurons were still in a rather early developmental state, whereas the midbrain-derived TH-ir neurons were more mature. Moreover, the increased number of TH/MAP2-ir neurons in the low oxygen cultures suggests an important effect of reduced oxygen tension on maturation of the cells. This is supported by a study by Liu et al. in 2009 on human mesencephalic progenitors, in which an accelerated maturation of TH expressing neurons was seen for cells grown at low as compared to high oxygen tension. Surprisingly, low oxygen tension did not increase the number of TH-ir cells in their cultures [19]. This discrepancy is likely to be explained by the time of exposure to low oxygen. In the present study cultures were grown at low oxygen tension during both expansion and differentiation, whereas Liu et al. only exposed the cultures to low oxygen tension during their differentiation [19].

Cells were also stained by double immunofluorescence for TH and GABA, and as expected the midbrain-derived cells did not display any co-localization. In the forebrain cultures, however, several of the TH-ir cells co-expressed GABA, although the majority of the TH-ir neurons did not. This was in accordance with a previous study showing that most forebrain stem cells differentiated into dopaminergic neurons with a midbrain phenotype, whereas only some co-expressed TH and GABA, presumable resembling the phenotype of dopaminergic interneurons of the olfactory bulb [34].

Western blot analyses revealed increased levels of β-tub III in both midbrain and forebrain cultures when differentiated at low as compared to high oxygen tension, and this level was highest for midbrain-derived cells. In contrast, the level of GFAP was higher for forebrain than midbrain cells, and for both types of cells enhanced when exposed to low oxygen tension. This was in contrast to Milosevic et al. (2005) who found an up-regulation of GFAP in murine mesencephalic NPCs when cultured at high as compared to low oxygen tension [48]. Contrary to our data, this research group also found a significantly lower number of mature MAP2-ir neurons in cultures grown at low oxygen tension. Possible explanations for this discrepancy may relate to the culture method applied (neurospheres versus adherent cultures), the use of primary cells instead of immortalized stem cell lines, use of different propagation and differentiation protocols, as well as species specific differences between mouse and human tissue.

Our data on Western blotting and immunocytochemistry for GFAP and β-tub III may indicate that the forebrain cells are more determined toward an astroglial fate, whereas the midbrain cells mainly form neurons. This could reflect differences in neuronal and glial differentiation capacity or region-specific requirements of the NSCs. In favour of this hypothesis is the fact that we developed our dopaminergic differentiation protocol using the midbrain-derived cell line [18] and with the aim of generating authentic and functional dopaminergic neurons with A9 (substantia nigra) characteristics [39], [47]. Thus, the applied protocol may not be optimal, when it comes to guiding NSCs from other brain regions towards the A9 dopaminergic phenotype.

### Low oxygen tension – mechanism of action

QPCR analysis showed significantly higher expression of vascular endothelial growth factor (VEGF) mRNA when midbrain cultures were differentiated at low as compared to high oxygen tension. Moreover, a strong tendency of increased erythropoietin (EPO) mRNA expression was found in both midbrain and forebrain cultures grown at low as compared to high oxygen tension. Surprisingly, the expression of Hypoxia Inducible Factor (HIF)-1alpha mRNA appeared independent of oxygen tension. One explanation could be that HIF-1alpha is mainly regulated at protein level through degradation of the protein. This degradation requires oxygen and during high oxygen conditions HIF-1alpha is polyubiquitinated by an E3 ubiquitin ligase complex and targeted for degradation [53], [54]. However, at oxygen levels below 5% HIF-1alpha begins to stabilize, due to inhibition of the E3 ubiquitin ligase complex, and starts to activate the transcription of HIF-1alpha target genes such as EPO and VEGF [13]. In accordance with this we found an upregulation of HIF-1alpha protein by Western blotting in midbrain cultures differentiated at low as compared to high oxygen [18]. HIF-1alpha, but also EPO and VEGF have previously been shown to exert neuroprotective effects on midbrain dopaminergic neurons [29], [31]-[33]. In 2007, Milosevic et al. reported that midbrain-derived neural precursor cells from conditional HIF-1alpha knockout mice displayed an impaired proliferative capacity and ability to survive. Furthermore, the dopaminergic differentiation potential of these cells was markedly reduced. Interestingly, treatment of HIF-1alpha knockout mNPCs with VEGF partially recovered both their proliferation and the dopaminergic differentiation capacity [29].

### Cell death and cell proliferation

Excessive oxygen (hyperoxic condition) is well known to increase cell death *in vitro* [55], and previous studies have suggested region-specific variations in oxygen sensitivity for both developing murine [48] and human brain tissue [21].

In our cultures cell death was investigated by immunostaining for active Caspase3 (marker of apoptotic cells) and analysis of LDH released to the culture medium. No significant differences were found in the relative content of Caspase3-ir cells between the cell lines, which was not surprising given that both cell lines are overexpressing the anti-apoptotic protein Bcl-X_L_ that is known to inhibit Caspase3-dependent apoptosis [56]. This observation was also in accordance to our previous study in which Bcl-X_L_ was found to significantly reduce the extent of cell death when compared to the naïve midbrain NSCs without Bcl-X_L_ overexpression [18]. In contrast, we found a significantly increased LDH release throughout the differentiation period for both midbrain and forebrain cells when cultured at high as compared to low oxygen tension. Cell viability, measured by MTS reduction, was also found to be significantly higher in midbrain and forebrain cultures, when grown at low oxygen tension. Taken together our results suggest that the cell death induced by hyperoxic conditions does not solely work through apoptosis, but also involves cell death mechanisms that cannot be prevented by Bcl-X_L_. The observation that cell death in both midbrain and forebrain NSC cultures was increased at high oxygen tension is in contrast to a previous study in which only midbrain precursor cells were hampered by a hyperoxic environment [48].

Besides stimulating the dopaminergic differentiation and reducing cell death in NPCs, low oxygen tension has also been found to increase cell proliferation as described above [17], [21]–[23], [48]. In addition we quantified the number of proliferating Ki67-ir cells in differentiating cultures, and found a high proportion of these cells in all cultures. In midbrain cultures, the number of dividing cells did not differ between low and high oxygen tension, whereas the number of dividing cells was significantly higher in forebrain cells when cultured at low oxygen as compared to high oxygen. The relatively high number of dividing cells in all cultures suggests that the cultures had not yet reached their full differentiation potential, hence it may still be possible to induce more cells to become dopaminergic and thereby increase the dopaminergic cell pool.

### Microtransplantation of human NSCs into striatal slice cultures

We have established a simple slice culture model of cell grafting into the dopamine-depleted striatum allowing screening of donor cells and development of new strategies for improvement of their survival and structural integration. Here we used this model to investigate whether our pre-differentiated (4 DIV) human NSCs were able to survive and differentiate into dopaminergic neurons after microtransplantation into mouse striatal brain tissue cultured at high or low oxygen tension.

For both cell lines we found significant numbers of HN-ir cells in the striatal slices when cultured at low oxygen tension. Whereas the number of midbrain-derived HN-ir cells in the striatal slices was 5–7 times higher than the number of cells transplanted, the number of forebrain-derived HN-ir cells recovered in the striatal slices cultures was almost identical to the number of exogenous cells transplanted. Lowering the oxygen tension during culturing of the transplanted striatal brain slices also significantly increased the relative content of human TH-ir cells in the brain slices.

The extent of migration and length of the neuronal processes was found to be more pronounced when midbrain transplanted cultures were grown at low oxygen, further supporting low oxygen as an important inducer of midbrain dopaminergic neuronal differentiation. The density of TH-ir cells in the midbrain transplanted cultures was higher compared to the forebrain transplanted cultures. In addition, the midbrain-derived TH-ir cells had a very mature neuronal appearance whereas the forebrain-derived TH-ir cells displayed an immature neuronal morphology. One explanation of these differences could be that even though these cells are multipotent [18], [37], [47] and respond to overlapping growth and differentiation factors, they are not equivalent to each other and maintain certain region-specific properties. Consequently they respond differently to local environmental factors released from the surrounding host tissue microenvironment. This hypothesis is supported by the study of Nishino et al. (2000) who showed that following bilateral intrastriatal grafting of pre-differentiated rodent mesencephalic or cortical NPCs into hemiparkinsonian rats, only the mesencephalic cells differentiated into TH-ir cells and ameliorated the amphetamine-induced rotation behavior. Interestingly they also found an increased differentiation of the mesencephalic-derived cells into TH-ir cells in the dopamine-depleted striatum compared to the intact striatum [57]. Taken together these results indicate that the DA-depleted striatum offers a suitable environment for midbrain-derived NSCs, but not necessarily for NSCs from other brain areas.

## Conclusion

In conclusion, our findings demonstrate that modulation of oxygen tension is a recognizable factor for the in vitro propagation and dopaminergic differentiation of human neural stem cells. The observed differential effect of oxygen on stem cells of midbrain and forebrain origin is likely to reflect differences in dopaminergic differentiation capacity or requirements of cells originating from brain regions with different microenvironments (stem cell niches).
